# *Glehnia littoralis* Root Extract Inhibits Fat Accumulation in 3T3-L1 Cells and High-Fat Diet-Induced Obese Mice by Downregulating Adipogenic Gene Expression

**DOI:** 10.1155/2018/1243049

**Published:** 2018-04-18

**Authors:** Heeok Hong, Joseph F. dela Cruz, Won Seob Kim, Kiyeol Yoo, Seong Gu Hwang

**Affiliations:** ^1^Department of Medical Science, School of Medicine, Konkuk University, Seoul 05029, Republic of Korea; ^2^Department of Animal Life and Environmental Science, Hankyong National University, Anseong 17579, Republic of Korea; ^3^College of Veterinary Medicine, University of the Philippines, Los Baños, Philippines; ^4^Department of Animal Science and Technology, Konkuk University, Seoul 05029, Republic of Korea; ^5^Department of Biological Sciences, Dankook University, Cheonan 31116, Republic of Korea

## Abstract

*Glehnia littoralis* has been reported to have several pharmacological properties but no reports describing the antiadipogenic effect of this plant have been published. This study was conducted to investigate the effects of* Glehnia littoralis* root hot water extract (GLE) and its underlying mechanism on 3T3-L1 cell adipogenesis and in high-fat diet- (HFD-) induced obese mice. We measured intracellular lipid accumulation using oil red O staining* in vitro*. For* in vivo* study, twenty-eight C57BL/6J male mice were randomly divided into four groups, Control, HFD, HFD + 1% GLE, and HFD + 5% GLE, which was performed for eight weeks. We determined the expression levels of the adipogenesis-related proteins by RT-PCR and western blotting in HFD-induced obese mice. The GLE dose-dependently inhibited 3T3-L1 adipocyte differentiation and intracellular lipid accumulation in differentiated adipocytes. Further, body weight gain and fat accumulation were significantly lower in the GLE-treated HFD mice than in the untreated HFD mice. GLE treatment suppressed the expression of adipogenic genes such as peroxisome proliferator-activated receptor (PPAR) *γ*, CCAAT/enhancer-binding protein (C/EBP) *α*, fatty acid synthase (aP2), and fatty acid synthase (FAS). These results suggest that the GLE inhibits adipocyte differentiation and intracellular lipid accumulation by downregulating the adipogenic gene expression both* in vitro* and* in vivo*.

## 1. Introduction

The prevalence of obesity has increased dramatically worldwide owing to lifestyle and diet changes and is rapidly becoming a threat to human health. Obesity has recently attracted increasing attention owing to its association with several metabolic diseases including type II diabetes, cardiovascular disease, and hypertension [1].

Obesity is caused by excess adipose tissue mass, which is the major energy reserve in the body [2]. As the adipose tissue mass can be modulated by inhibiting adipogenesis (differentiation of preadipocytes to mature adipocytes) [3], obesity treatments are usually targeted at suppressing energy or food intake, preadipocyte differentiation and proliferation, and lipogenesis, while increasing energy expenditure, lipolysis, and fat oxidation [4]. However, no effective treatment options are currently available for obesity. Therefore, plant-based bioactive materials are being isolated and their pharmacological properties are being actively researched [5, 6]. Several studies suggest that phytochemical treatments can regulate adipose tissue mass by inhibiting adipogenesis [3, 7, 8].

*Glehnia littoralis *Fr. Schmidt ex Miq. (Umbelliferae) is a perennial herb that grows on the sandy beaches of eastern China, Korea, Japan, and North-west America [9]. Its roots and rhizomes, which are listed in the Korean, Chinese, and Japanese Pharmacopoeias [10], have traditionally been used for their diaphoretic, antipyretic, antiphlogistic, and analgesic properties. Further, the aqueous extract of* G. littoralis* has been reported to have several pharmacological properties including antioxidant [11], anticancer [12, 13], anti-inflammatory [10], and some immunomodulatory properties [14, 15]. The major components of the underground parts of* G. littoralis* have been identified as quercetin, isoquercetin, rutin, chlorogenic acid, and caffeic acid [11].

To date, no reports describing the antiadipogenic effect of this plant have been published. High-fat diet- (HFD-) induced animal models of obesity and 3T3-L1 cells have been widely used for studying the antiobesity properties of various compounds [16]. Therefore, this study was conducted to elucidate the effects of the* Glehnia littoralis* root extract (GLE) on the adipogenic differentiation of 3T3-L1 cells by measuring intracellular lipid accumulation. We also investigated the mechanism underlying the inhibitory effects of GLE on adipocyte differentiation in HFD-induced obese mice to determine the potential medicinal benefits of* G. littoralis *as an antiobesity agent.

## 2. Materials and Methods

### 2.1. Preparation of* Glehnia littoralis* Root Extract (GLE)

*G. littoralis* roots obtained from Fine Food Tech Co., Ltd. (Gongju, Korea), were air-dried at 50°C at an air velocity of 1.5 m/s for 4 days, blended, and further ground, to obtain a fine powder. The powder (300 g) was soaked in 3 L of distilled water and then heated at 100°C for 4 h. The crude extract was collected, filtered with a sterilized cloth, freeze-dried at −60°C, and stored in a deep freezer (−70°C) until use.

### 2.2. Determination of the Polyphenol Components of GLE by High-Performance Liquid Chromatography (HPLC)

The HPLC analysis was performed on a Dionex Summit™ system (Thermo Scientific, Waltham, MA, USA) equipped with an UVD 340U-photodiode array detector (Dionex, Sunnyvale, CA, USA) using a reverse-phase C18 analytical column (4.6 × 250 mm i.d., 5 *µ*m, Shiseido Capcell Pak MG). The mobile phase was solvent A (methanol, acetic acid, and water at 10 : 2 : 88 v/v/v) and solvent B (methanol, acetic acid, and water at 90 : 3 : 7 v/v/v). The analysis was performed under the following gradient conditions: 100% A to 0% B (0–30 min), 100% B (30–40 min), 100% B to 0% A (40–42 min), and 100% A (42–60 min), with a flow rate of 1 mL/min and a detection wavelength of 280 nm with 1 nm bandwidth. All standards were purchased from Sigma-Aldrich (St. Louis, MO, USA).

### 2.3. Cell Culture and Differentiation

Murine 3T3-L1 preadipocytes were obtained from the Korean Cell Bank (Seoul, Korea) and cultured to confluence in Dulbecco's modified Eagle's medium (DMEM, Gibco, Rockville, MD, USA) supplemented with 10% fetal bovine serum (FBS, Gibco, Rockville, MD, USA) and 1% penicillin-streptomycin (Gibco, Rockville, MD, USA) in a humidified 5% CO_2_ atmosphere at 37°C. On day 2 after confluence (designated as day 0), cell differentiation was induced with the MDI differentiation medium containing 1 *µ*M dexamethasone (DEX, Sigma-Aldrich, St. Louis, MO, USA), 0.5 mM 3-isobutyl-1-methylxanthine (IBMX, Sigma-Aldrich, St. Louis, MO, USA), 10 *µ*g/mL insulin (INS, Sigma-Aldrich, St. Louis, MO, USA), and DMEM supplemented with 10% FBS. After 48 h (day 2), the culture medium was replaced with DMEM supplemented with 10% FBS, and this was repeated every 48 h until day 8. The cells were treated with different concentrations of the GLE (0, 50, 100, 200, and 400 *µ*g/mL) from day 0 to 8, and untreated cells were used as a control.

### 2.4. Determination of Cell Viability

The effect of different concentrations of the GLE on 3T3-L1 preadipocyte viability was determined by the cell counting kit-8 (CCK-8) assay (Dojindo Molecular Technologies, Tokyo, Japan). Briefly, the cells were seeded in a 96-well plate at a density of 1 × 10^4^ cells/well and treated with the GLE (0–400 *µ*g/mL) for 24 h. 10 *µ*L of CCK-8 reagent was then added to each well and the absorbance was measured at 450 nm using an Infinite® F50 microplate reader (Tecan, Männedorf, Switzerland). The viability of the GLE-treated cells was expressed as a percentage of the control cell viability.

### 2.5. Oil Red O Staining and Estimation of the Intracellular Lipid Content

The lipid accumulation in adipocytes, which indicates the extent of differentiation, was measured using oil red O staining. Briefly, differentiated 3T3-L1 cells were fixed in 10% formaldehyde in PBS for 1 h, washed with distilled water, and dried completely. The cells were then stained with 0.5% oil red O solution in 60 : 40 (v/v) isopropanol : triple distilled water for 15 min at room temperature, washed four times with triple distilled water, and dried. The treated cells were observed under an Olympus microscope (BX51, Tokyo, Japan), and representative images were captured using an Olympus DP70 camera. The cell differentiation was quantified by elution of the stain with isopropanol and measurement of the absorbance at 520 nm.

### 2.6. Animals and Diets

C57BL/6J male mice (6- to 8-week-old) were purchased from Samtako Bio Korea Co., Ltd., (Osan, Korea) and initially acclimated to laboratory conditions for 1 week, prior to experimental use. After acclimatization, 28 mice were randomly divided into four groups, namely, the American Institute of Nutrition- (AIN-) 93G diet (control, C), high-fat diet (HFD), HFD with 1% GLE (HFD + 1% GLE), and HFD with 5% GLE (HFD + 5% GLE) groups. The HFD contained 45.5% fat (as soybean oil and lard), 20% protein, and 34.5 % carbohydrate (Table 1).

The mice were housed under a 12 : 12 h light-dark cycle at 22 ± 2°C and 55 ± 5% relative humidity with ad libitum access to the specified diets and sterile drinking water for 8 weeks. The food intake and body weight were measured every week, and the feed efficiency ratio (FER) was calculated as the total weight gain/total food intake. All experiments on animals were carried out in accordance with the institutional guidelines of the Hankyong National University, Anseong, Korea. This study conformed to the Guide for the Care and Use of Laboratory Animals published by the US National Institutes of Health (NIH publication number 85-23, revised 1996, latest revision in 2011), and was approved by the Hankyong National University Animal Welfare Committee (Hankyong. 2015-2).

At the end of the experimental period, the animals were fasted overnight and administered mild ether anesthesia, and blood was collected via puncture of the retroorbital sinus in ethylenediaminetetraacetic acid- (EDTA-) coated vials. The animals were then euthanized by cervical dislocation under mild ether anesthesia and the abdominal, perirenal, and epididymal fat pads were excised. The fat samples were rinsed with saline and stored at −70°C until further analysis.

### 2.7. RNA Extraction and Reverse Transcription-Polymerase Chain Reaction (RT-PCR)

Total RNA was isolated from the epididymal fat samples of the experimental mice using the RNAiso Plus reagent (Takara Bio Inc., Shiga, Japan) according to the manufacturer's instructions. cDNA was synthesized from 1 *µ*g of the total RNA in a 20 *µ*L reaction volume using a Maxime RT PreMix kit (iNtRON Biotechnology, Seongnam, Korea) containing the OptiScript™ reverse transcriptase and i-StarTaq™ DNA polymerase, following the manufacturer's recommended protocol. The oligonucleotide primers are shown in Table 2. The PCR conditions consisted of an initial denaturation step at 95°C for 5 min, followed by 30 amplification cycles consisting of denaturation for 40 s at 95°C, annealing for 40 s (temperature 56–62°C), and extension for 1 min at 72°C. The PCR products were separated on an agarose gel (1.5%) by electrophoresis for 30 min at 100 V. The bands were visualized, and their relative intensities were analyzed using the ImageJ software (National Institutes of Health, Bethesda, MD, USA).

### 2.8. Western Blot Analysis

Proteins were extracted from the epididymal fat samples using a protein extraction kit (iNtRON Biotechnology, Seongnam, Korea). The lysates were centrifuged at 15,000 rpm for 15 min at 4°C, and the protein content of the supernatant was determined by Bio-Rad™ assay kit (Hercules, CA, USA). Diluted protein samples (30 *µ*g) were separated by sodium dodecyl sulfate-polyacrylamide gel electrophoresis (SDS-PAGE, 10%) and transferred to nitrocellulose membranes. The membranes were blocked overnight with 5% skim milk in Tris-buffered saline-Tween 20 (TBST, 20 mM Tris-HCl, pH 7.6, 140 mM NaCl, and 0.1% Tween 20) and incubated with the following primary antibodies (1 : 1000 dilution): PPAR*γ*, C/EBP*α*, SREBP-1c, aP2, leptin, FAS, and *β*-actin (Abcam, Cambridge, UK). The membranes were then washed four times with TBST buffer and incubated with the corresponding horseradish-peroxidase- (HRP-) conjugated secondary antibody (1 : 2000 dilution). The immunoreactive protein bands were visualized using an enhanced chemiluminescence plus kit (Amersham Pharmacia Biotech, Buckinghamshire, UK), and their relative intensities were quantified using the ImageJ 1.41 software.

### 2.9. Statistical Analysis

The results are expressed as the mean ± standard deviation (SD) of at least three independent experiments. Statistical differences between the groups were evaluated by one-way analysis of variance (ANOVA) followed by Duncan's multiple range test. Values of *p* < 0.05 were considered statistically significant. The statistical analysis system (SAS) software package version 9.2 (SAS Institute Inc., Cary, NC, USA) was used for the analysis.

## 3. Results and Discussion

### 3.1. Determination of Active Components of GLE

**Figure 1:**
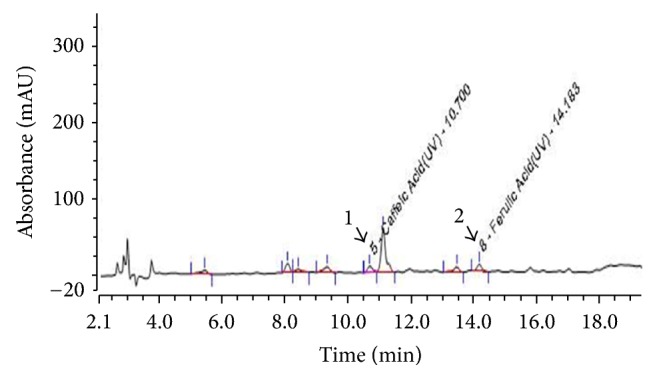
*HPLC profile and chemical structures of the polyphenol components of the Glehnia littoralis root extract (GLE)*. Caffeic acid (peak 1) and ferulic acid (peak 2).

When the composition of the GLE was investigated by comparing its HPLC profile with that of nine standard compounds including cnidilide, ligustilide, neocnidilide, butylphthalide, senkyunolide, tetramethylpyrazine, caffeic acid, ferulic acid, and perlolyrine eluted under the same conditions, two compounds, namely, caffeic acid and ferulic acid, were identified as the active constituents of the GLE (Figure 1).

### 3.2. Effect of the GLE on 3T3-L1 Cell Proliferation

**Figure 2:**
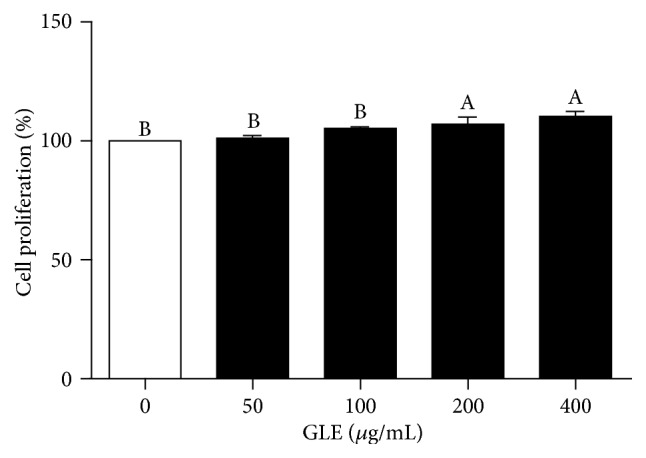
*Effect of the GLE on 3T3-L1 cell proliferation*. 3T3-L1 preadipocytes were cultured in serum-free medium with GLE (0–400 *μ*g/mL) for 24 h. Posttreatment cell viability was determined by cell counting kit- (CCK-) 8 assay. Values are expressed as mean ± SD (*n* = 3). Viability of untreated controls is set to 100%. Means with different superscript letters are significantly different by Duncan's multiple range test (*p* < 0.05). GLE,* Glehnia littoralis* root extract.

The cytotoxicity of the GLE was evaluated prior to the investigation of its antiadipogenic effects on 3T3-L1 cells. Treatment with different concentrations (50–400 *µ*g/mL) of the GLE for 24 h stimulated the proliferation of 3T3-L1 cells with no cytotoxicity observed following the treatment with 400 *µ*g/mL of the GLE for 24 h (Figure 2).

### 3.3. Effect of the GLE on 3T3-L1 Preadipocytes Differentiation

**Figure 3:**
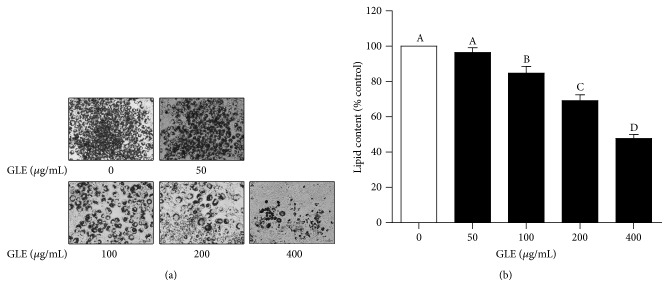
*Effect of the GLE on 3T3-L1 adipocyte differentiation*. (a) Oil red O staining showing the differentiation of induced 3T3-L1 preadipocytes. Black color indicates stained cytoplasmic lipids. (b) Quantification of lipid accumulation in differentiated 3T3-L1 cells based on the absorbance at 520 nm of destained oil red O extracted from the adipocytes. Lipid content in untreated control cells is set to 100%. Values are expressed as mean ± SD (*n* = 3). Means with different superscript letters are significantly different by Duncan's multiple range test (*p* < 0.05). GLE,* Glehnia littoralis* root extract.

We evaluated the effect of the GLE on postconfluent 3T3-L1 preadipocytes that were induced to differentiate in MDI differentiation medium for 2 days. Oil red O staining was used to monitor the changes in lipid accumulation during preadipocyte differentiation. Representative images of the oil red O-stained, GLE-treated cells acquired on day 8 of the differentiation period showed a dose-dependent suppression of intracellular lipid accumulation (Figures 3(a) and 3(b)). The lipid content decreased by 31 and 52% in response to 200 and 400 *µ*g/mL of the GLE, respectively. Adipogenesis, the stage of the cell differentiation process during which preadipocytes mature into adipocytes, is accompanied by lipid accumulation as well as changes in gene expression and hormone sensitivity [17]. These results show the inhibitory effect of the GLE on adipocyte differentiation.

### 3.4. Effect of the GLE in HFD-Induced Obese Mice

**Figure 4:**
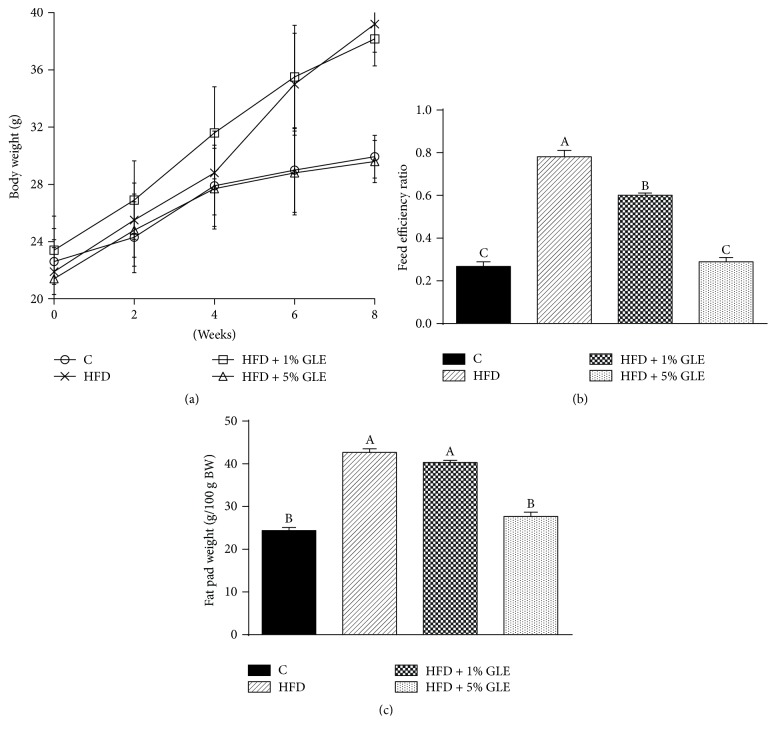
*Effect of the GLE on the growth of and fat accumulation in HFD-induced obese mice*. (a) Body weight of the mice that were fed experimental diets. (b) Feed efficiency ratio (FER) calculated as the total weight gain/total food intake. (c) Fat weight per 100 g body weight. Fat weight includes the abdominal, renal, and epididymal fat pad weights of mice that were fed experimental diets. Values are presented as mean ± SD (*n* = 7). Each bar with different superscript letters is significantly different by Duncan's multiple range test (*p* < 0.05). Experimental groups: Control, fed basic diet; HFD, fed high-fat diet; HFD + 1% GLE, fed HFD containing 1% GLE; HFD + 5% GLE, fed HFD containing 5% GLE. GLE,* Glehnia littoralis* root extract; HFD, high-fat diet.

We further elucidated the antiadipogenic effects of the GLE by performing an* in vivo* experiment with HFD-induced obese mice. As shown in Figure 4(a), the body weights of mice in the HFD and HFD + 1% GLE groups were significantly higher than those of mice in the control and HFD + 5% GLE groups after 6 weeks of treatment (*p* < 0.05). At the end of the experiment, mice in the HFD + 5% GLE group exhibited a drastic reduction in body weight gain compared to that reported for the HFD group mice (8.2 ± 3.4 versus 17.3 ± 2.6 g). However, the antiadipogenic effect in the HFD + 1% GLE group was not as pronounced as that in the HFD + 5% GLE group. The feed efficiency ratio (FER) of the HFD + 5% GLE group was significantly lower than that of the HFD and HFD + 1% GLE groups (Figure 4(b)) (*p* < 0.05). The fat weight, which comprises the abdominal, perirenal, and epididymal fat pad weights, of mice in the HFD + 5% GLE group (8.2 ± 0.3 g) was approximately 50% lower than that of mice in the HFD (16.3 ± 0.3 g) and HFD + 1% GLE (15.8 ± 0.2 g) groups. The fat weight per 100 g body weight of mice in the HFD + 5% GLE group (27.7 ± 1.0 g) was significantly lower than that of mice in the HFD (42.7 ± 0.8 g) and HFD + 1% GLE (40.3 ± 0.5 g) groups (Figure 4(c)) (*p* < 0.05).

It is well-known that an imbalance between energy intake and energy expenditure leads to body fat storage owing to increased lipogenesis and adipogenesis [18]. However, this study showed that supplementing the diet with 5% GLE effectively inhibited the body fat accumulation in HFD-induced obese mice compared with that in the untreated HFD group. Therefore, the GLE could be useful for treating obesity by reducing body fat accumulation.

### 3.5. Effects of the GLE on Critical Adipogenic Gene and Protein Expression in HFD-Induced Obese Mice

**Figure 5:**
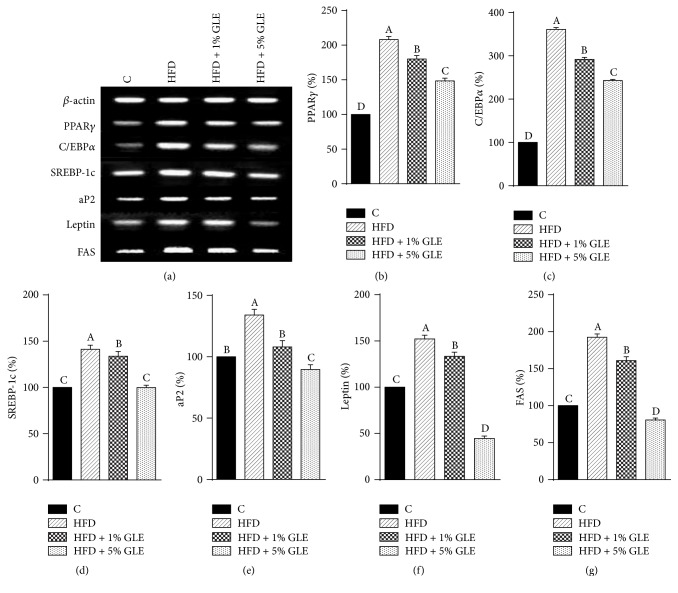
*Effect of the GLE on the mRNA expression of major adipogenic transcription factors in HFD-induced obese mice*. (a) A representative image of the RT-PCR results. mRNA levels of (b) PPAR*γ*, (c) C/EBP*α*, (d) SREBP-1c, (e) aP2, (f) Leptin, and (g) FAS as determined by RT-PCR. Values are presented as a percentage of the levels in controls. Data are expressed as mean ± SD (*n* = 7). Bars with different superscript letters are significantly different by Duncan's multiple range test (*p* < 0.05). Experimental groups: Control, fed basic diet; HFD, fed high-fat diet; HFD + 1% GLE, fed HFD containing 1% GLE; HFD + 5% GLE, fed HFD containing 5% GLE. PPAR, peroxisome proliferator-activated receptor; C/EBP, CCAAT/enhancer-binding protein; FAS, fatty acid synthase; aP2, adipose fatty acid binding protein; SREBP, sterol regulatory element binding protein; RT-PCR, reverse transcription-polymerase chain reaction; GLE,* Glehnia littoralis* root extract; HFD, high-fat diet.

In order to investigate the molecular mechanisms underlying the antiadipogenic effect of the GLE in HFD-induced obese mice, we analyzed the gene and protein expression of various transcription factors associated with preadipocyte differentiation and fat accumulation via RT-PCR and western blotting, respectively. The GLE treatment markedly decreased the expression of adipogenic markers such as PPAR*γ*, C/EBP*α*, and SREBP-1c and lipid metabolism genes such as aP2, leptin, and FAS (Figure 5). The mRNA levels of PPAR*γ*, C/EBP*α*, and SREBP-1c in the GLE-treated groups were significantly lower than those in the HFD group (*p* < 0.05), with the levels in the HFD + 5% GLE group being reduced by 59.5, 118.3, and 41.3%, respectively, compared to those in the HFD group (Figures 5(b)–5(d)).

Preadipocyte differentiation is regulated by transcriptional activators including members of the C/EBP and PPAR*γ* families [19–21]. Currently, C/EBP*α* and PPAR*γ* are considered the primary mediators of adipogenesis. These transcription factors have been shown to activate adipocyte-specific genes and are also involved in the growth arrest required for preadipocyte differentiation [22]. The complex process of adipogenesis commences with PPAR*γ* production, which is controlled and activated by C/EBP*α* and SREBP-1c [17]. C/EBP*α* also activates the promoters of the adipocyte genes leptin and aP2 [23], while both PPAR*γ* and C/EBP*α* coordinate the expression of genes involved in generating and maintaining aP2 and leptin levels. The expression of aP2 and FAS mRNA in the HFD group was 134.1 ± 4.6% and 192.4 ± 4.6%, while that in the 5% GLE-treated group was 89.7 ± 3.9% and 80.7 ± 2.5%, respectively, compared to the expression in the control group (100%) (Figures 5(e) and 5(g)). The mRNA expression of leptin, which serves as a major adipostat by suppressing the urge to eat and promoting energy expenditure [24], decreased by 19 and 107.7% in a dose-dependent manner compared with that in the HFD group, following the treatment with 1 and 5% GLE, respectively (Figure 5(f)). Interestingly, the 5% GLE treatment significantly decreased the expression of aP2, leptin, and FAS mRNA, compared to the expression in the control group (*p* < 0.05). In particular, the leptin mRNA expression in the HFD + 5% GLE group decreased by 44.6 ± 2.7%. The GLE treatment also suppressed the expression of SREBP-1c and FAS. SREBP-1c accelerates adipogenesis by inducing the expression of FAS. Leptin, which is one of the best-known hormone markers for obesity, was also downregulated following the ingestion of an HFD with 5% GLE. These findings also indicate that GLE might contain FAS or leptin inhibitors and present its efficiency against fat accumulation through this pathway in addition to adipogenesis inhibition. It has been reported that caffeic acid phenethyl ester suppresses the production of leptin during differentiation of 3T3-L1 preadipocytes [25]. Therefore, one of components of GLE such as caffeic acid may be responsible inhibitor for both FAS and leptin.

**Figure 6:**
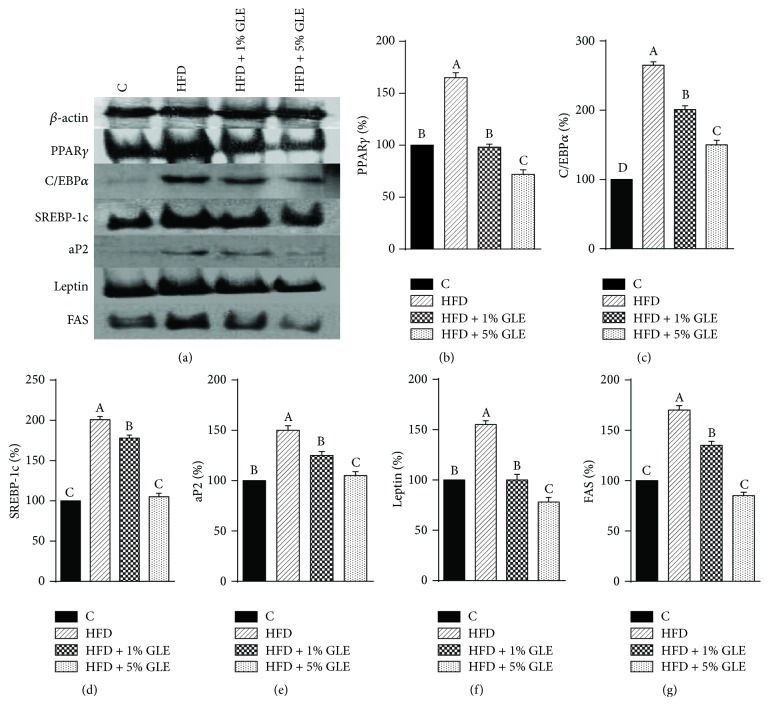
*Effect of the GLE on the protein expression of major adipogenic transcription factors in HFD-induced obese mice*. (a) A representative image of the western blotting results. Protein levels of (b) PPAR*γ*, (c) C/EBP*α*, (d) SREBP-1c, (e) aP2, (f) Leptin, and (g) FAS as determined by western blotting. Values are presented as a percentage of the levels in controls. Data are expressed as mean ± SD (*n* = 7). Bars with different superscript letters are significantly different by Duncan's multiple range test (*p* < 0.05). Experimental groups: Control, fed basic diet; HFD, fed high-fat diet; HFD + 1% GLE, fed HFD containing 1% GLE; HFD + 5% GLE, fed HFD containing 5% GLE. PPAR, peroxisome proliferator-activated receptor; C/EBP, CCAAT/enhancer-binding protein; FAS, fatty acid synthase; aP2, adipose fatty acid binding protein; SREBP, sterol regulatory element binding protein; GLE,* Glehnia littoralis* root extract; HFD, high-fat diet.

PPAR*γ* and C/EBP*α* are major regulators of the preadipocyte differentiation process, and C/EBP*α* mediates the transactivation of leptin transcription [26]. C/EBP*α*, which is expressed rather late in the adipogenesis process, has been widely reported to be both necessary and sufficient for the differentiation of 3T3-L1 preadipocytes to adipocytes [23, 27, 28] and appears to promote the differentiation in conjunction with PPAR*γ* by cross-regulation [29]. SREBP-1c regulates the lipogenic gene expression associated with fatty acid synthesis, which promotes increased triglyceride synthesis and the expression of PPAR*γ* ligands [30]. The results of our study suggest that the GLE downregulates the expression of SREBP-1c, leading to decreased PPAR*γ* expression. SREBP-1c also reportedly binds to the promoter region of FAS to activate its transcription [31]. The expression of aP2 and FAS genes, which are involved in lipid metabolism, was significantly downregulated in the GLE-treated HFD mice. aP2, which is expressed in adipocytes and is also known as the fatty acid binding protein 4 (FABP4), has profound effects on insulin sensitivity and glucose metabolism and plays an important role in adipocyte differentiation [32]. Additionally, aP2 is activated by PPAR*γ*, C/EBP*α*, and SREPB-1c [32]. Furthermore, the protein levels of the adipogenic transcription factors and lipid metabolism genes, namely, PPAR*γ*, C/EBP*α*, SREPB-1c, aP2, leptin, and FAS, in the epididymal fat of the GLE-treated HFD mice followed the same trend as their respective mRNA levels (Figures 6(a)–6(g)). Thus, the expression of the critical adipogenic proteins PPAR*γ* and C/EBP*α* decreased following the treatment with 1 and 5% GLE (Figures 6(b) and 6(c)). In connection with the discussion before, it has been suggested that GLE might suppressed the secretion of adipocytokines such as leptin through the suppression of PPAR*γ* expression [25].

Obesity is related to adipocyte differentiation and excess fat accumulation [18]. In our study, GLE administration reduced fat accumulation in 3T3-L1 adipocytes and HFD-induced obese mice by suppressing the expression of key transcription factors and genes at both the mRNA and protein level. SREBP-1c is known to accelerate adipogenesis by inducing the expression of FAS, which is an adipogenic enzyme [33]. Additionally, triglyceride accumulation in the livers of SREBP-1c-deficient ob/ob mice has been reported to decrease by approximately 50% compared with that in ob/ob mice livers [34].

Our results showed that the abdominal, perirenal, and epididymal fat weight of 5% GLE-treated mice was less than half of that of the untreated HFD-induced obese mice, which may have been due to the GLE-mediated inhibition of the mRNA and protein expression of SREBP-1c and FAS. We also demonstrated that the antiobesity effects of the GLE on various genes involved in adipogenesis, which is a differentiation pathway, are mediated via the downregulation of major transcription factors including PPAR*γ*, C/EBP*α*, and SREBP-1c. The consequent downregulation of lipid metabolizing mediators such as aP2, leptin, and FAS, which are involved in the transport, uptake, and synthesis of lipids, resulted in the reduced fat accumulation in adipocytes.

## 4. Conclusion

In conclusion, the GLE strongly inhibited adipogenesis by reducing the expression of adipogenesis-related transcription factors. Therefore, the GLE may act as an effective nutraceutical for the treatment of obesity by suppressing either adipocyte differentiation or lipid accumulation.

## Figures and Tables

**Table 1:** Composition of experimental diets.

Ingredient	HFD	HFD + 1% GLE	HFD + 5% GLE
Casein	23.31	23.31	23.31
Sucrose	20.14	20.14	20.14
Dextrose	11.65	11.65	11.65
Corn starch	8.48	7.48	3.48
Cellulose	5.83	5.83	5.83
Soybean oil	2.91	2.91	2.91
Lard	20.69	20.69	20.69
Mineral mix^(1)^	5.24	5.24	5.24
Vitamin mix^(1)^	1.17	1.17	1.17
L-Cysteine	0.35	0.35	0.35
Choline bitartrate	0.23	0.23	0.23
GLE^(2)^		1.00	5.00

HFD: high-fat diet, HFD + 1% GLE: HFD containing 1% *Glehnia littoralis* root extract (GLE); HFD + 5% GLE: HFD containing 5% GLE. ^(1)^Mineral and vitamin mixtures were based on the AIN-93 standard diet for rodents. ^(2)^*Glehnia littoralis* root extract powder.

**Table 2:** List of primers used in RT-PCR analysis.

Gene	Forward primer	Reverse primer
PPAR*γ*	GATGGAAGACCACTCGCATT	AACCATTGGGTCAGCTCTTG
C/EBP*α*	TGGACAAGAACAGCAACGAG	TCACTGGTCAACTCCAGCAC
SREBP-1c	GCTGTTGGCATCCTGCTATC	TAGCTGGAAGTGACGGTGGT
aP-2	TCAGCGTAAATGGGGATTTGG	GTCTGCGGTGATTTCATCGGA
FAS	CCCTTGATGAAGAGGGATCA	ACTCCACAGGTGGGAACAAG
Leptin	TGAGTTTGTCCAAGATGGACC	GCCATCCAGGCT CTCTGG
*β*-Actin	CAC CCC AGC CAT GTA CGT	GTCCAGACGCAGGATGGC

## Abbreviations

GLE: * Glehnia littoralis* root extract
HFD: High-fat diet
PPAR*γ*: Peroxisome proliferator-activated receptor *γ*
C/EBP*α*: CCAAT/enhancer-binding protein *α*
FAS: Fatty acid synthase
aP2: Adipose fatty acid binding protein
SREBP-1c: Sterol regulatory element binding protein-1c.

## References

[1] Pagotto U., Vanuzzo D., Vicennati V., Pasquali R. (2008). Pharmacological therapy of obesity. *Giornale Italiano Di Cardiologia*.

[2] Couillard C., Mauriège P., Imbeault P. (2000). Hyperleptinemia is more closely associated with adipose cell hypertrophy than with adipose tissue hyperplasia. *International Journal of Obesity*.

[3] Yang J.-Y., Della-Fera M. A., Rayalam S. (2008). Enhanced inhibition of adipogenesis and induction of apoptosis in 3T3-L1 adipocytes with combinations of resveratrol and quercetin. *Life Sciences*.

[4] Yoon S.-S., Rhee Y.-H., Lee H.-J. (2008). Uncoupled protein 3 and p38 signal pathways are involved in anti-obesity activity of Solanum tuberosum L. cv. Bora Valley. *Journal of Ethnopharmacology*.

[5] Zacour A. C., Silva M. E., Cecon P. R., Bambirra E. A., Vieira E. C. (1992). Effect of dietary chitin on cholesterol absorption and metabolism in rats. *Journal of Nutritional Science and Vitaminology*.

[6] Kaplan L. M. (2005). Pharmacological therapies for obesity. *Gastroenterology Clinics of North America*.

[7] Lin J., Della-Fera M. A., Baile C. A. (2005). Green tea polyphenol epigallocatechin gallate inhibits adipogenesis and induces apoptosis in 3T3-L1 adipocytes. *Obesity Research*.

[8] Yang J.-Y., Della-Fera M. A., Hartzell D. L., Nelson-Dooley C., Hausman D. B., Baile C. A. (2006). Esculetin induces apoptosis and inhibits adipogenesis in 3T3-L1 cells. *Obesity*.

[9] Rozema J., Bijwaard P., Prast G., Broekman R. (1985). Ecophysiological adaptations of coastal halophytes from foredunes and salt marshes. *Plant Ecology*.

[10] Yoon T., Lee D. Y., Lee A. Y., Choi G., Choo B. K., Kim H. K. (2010). Anti-inflammatory effects of Glehnia littoralis extract in acute and chronic cutaneous inflammation. *Immunopharmacology and Immunotoxicology*.

[11] Yuan Z., Tezuka Y., Fan W., Kadota S., Li X. (2002). Constituents of the underground parts of Glehnia littoralis. *Chemical & Pharmaceutical Bulletin*.

[12] Kong C.-S., Um Y. R., Lee J. I., Kim Y. A., Yea S. S., Seo Y. (2010). Constituents isolated from Glehnia littoralis suppress proliferations of human cancer cells and MMP expression in HT1080 cells. *Food Chemistry*.

[13] Um Y. R., Kong C.-S., Lee J. I., Kim Y. A., Nam T. J., Seo Y. (2010). Evaluation of chemical constituents from Glehnia littoralis for antiproliferative activity against HT-29 human colon cancer cells. *Process Biochemistry*.

[14] Nakano Y., Matsunaga H., Saita T., Mori M., Katano M., Okabe H. (1998). Antiproliferative Constituents in Umbelliferae Plants IL1) Screening for Polyacetylenes in Some Umbelliferae Plants, and Isolation of Panaxynol and Falcarindiol from the Root of Heracleum moellendorffii. *Biological & Pharmaceutical Bulletin*.

[15] Ng T. B., Liu F., Wang H. X. (2004). The antioxidant effects of aqueous and organic extracts of Panax quinquefolium, Panax notoginseng, Codonopsis pilosula, Pseudostellaria heterophylla and Glehnia littoralis. *Journal of Ethnopharmacology*.

[16] Buettner R., Schölmerich J., Bollheimer L. C. (2007). High-fat diets: modeling the metabolic disorders of human obesity in rodents. *Obesity*.

[17] Ali A. T., Hochfeld W. E., Myburgh R., Pepper M. S. (2013). Adipocyte and adipogenesis. *European Journal of Cell Biology*.

[18] Spiegelman B. M., Flier J. S. (2001). Obesity and the regulation of energy balance. *Cell*.

[19] Farmer S. R. (2006). Transcriptional control of adipocyte formation. *Cell Metabolism*.

[20] Gregoire F. M., Smas C. M., Sul H. S. (1998). Understanding adipocyte differentiation. *Physiological Reviews*.

[21] Wu Z., Rosen E. D., Brun R. (1999). Cross-regulation of C/EBP*α* and PPAR*γ* controls the transcriptional pathway of adipogenesis and insulin sensitivity. *Molecular Cell*.

[22] White U. A., Stephens J. M. (2010). Transcriptional factors that promote formation of white adipose tissue. *Molecular and Cellular Endocrinology*.

[23] MacDougald O. A., Lane M. D. (1995). Transcriptional regulation of gene expression during adipocyte differentiation. *Annual Review of Biochemistry*.

[24] Wrann C. D., Rosen E. D. (2014). New insights into adipocyte-specific leptin gene expression. *Adipocyte*.

[25] Juman S., Yasui N., Okuda H. (2011). Caffeic acid phenethyl ester suppresses the production of adipocytokines, leptin, tumor necrosis factor -alpha and resistin, during differentiation to adipocytes in 3T3-L1 cells. *Biological & Pharmaceutical Bulletin*.

[26] Krempler F., Breban D., Oberkofler H. (2000). Leptin, Peroxisome Proliferator-Activated Receptor- , and CCAAT/Enhancer Binding Protein- mRNA Expression in Adipose Tissue of Humans and Their Relation to Cardiovascular Risk Factors. *Arteriosclerosis, Thrombosis, and Vascular Biology*.

[27] Lin F.-T., Lane M. D. (1992). Antisense CCAAT/enhancer-binding protein RNA suppresses coordinate gene expression and triglyceride accumulation during differentiation of 3T3-L1 preadipocytes. *Genes & Development*.

[28] Lin F.-T., Lane M. D. (1994). CCAAT/enhancer binding protein *α* is sufficient to initiate the 3T3-L1 adipocyte differentiation program. *Proceedings of the National Acadamy of Sciences of the United States of America*.

[29] Jeon T., Hwang S. G., Hirai S. (2004). Red yeast rice extracts suppress adipogenesis by down-regulating adipogenic transcription factors and gene expression in 3T3-L1 cells. *Life Sciences*.

[30] Kim J. B., Spiegelman B. M. (1996). ADD1/SREBP1 promotes adipocyte differentiation and gene expression linked to fatty acid metabolism. *Genes & Development*.

[31] Magaña M. M., Osborne T. F. (1996). Two tandem binding sites for sterol regulatory element binding proteins are required for sterol regulation of fatty-acid synthase promoter. *The Journal of Biological Chemistry*.

[32] Huang B., Yuan H. D., Kim D. Y., Quan H. Y., Chung S. H. (2011). Cinnamaldehyde prevents adipocyte differentiation and adipogenesis via regulation of peroxisome proliferator-activated receptor-*γ* (PPAR*γ*) and AMP-activated protein kinase (AMPK) pathways. *Journal of Agricultural and Food Chemistry*.

[33] Jung H.-Y., Kim Y.-H., Kim I.-B. (2013). The Korean mistletoe (*Viscum album coloratum*) extract has an antiobesity effect and protects against hepatic steatosis in mice with high-fat diet-induced obesity. *Evidence-Based Complementary and Alternative Medicine*.

[34] Yahagi N., Shimano H., Hasty A. H. (2002). Absence of sterol regulatory element-binding protein-1 (SREBP-1) ameliorates fatty livers but not obesity or insulin resistance in Lepob/Lepob mice. *The Journal of Biological Chemistry*.

